# Changes in renal function indices in cirrhotic chronic hepatitis C patients treated with sofosbuvir-containing regimens

**DOI:** 10.18632/oncotarget.18701

**Published:** 2017-06-28

**Authors:** Jianhong Chen, Xiaxia Zhang, Hao Luo, Chihong Wu, Min Yu, Dan Liu, Hongli Xi, Yihang Zhou, Yaoyu An, Xiaoyuan Xu

**Affiliations:** ^1^ Department of Infectious Disease, Peking University First Hospital, Beijing 100034, China

**Keywords:** chronic hepatitis C, directly acting antivirals, sofosbuvir, nephrotoxicity

## Abstract

This study aimed to explore changes in hepatic and renal function indices in chronic hepatitis C (CHC) patients treated with direct-acting antivirals (DAAs). Forty-three CHC patients treated with sofosbuvir (SOF)-containing regimens were enrolled. At the end of treatment, the estimated glomerular filtration rate (eGFR) level was significantly decreased and the serum creatinine (Scr) and uric acid (UA) levels were significantly increased compared with baseline levels (eGFR: 86.7 ± 20.4 vs 80.5 ± 21.3, *P*_01_ = 0.005; Scr: 83.9 ± 19.1 vs 89.6 ± 21.1, *P*_01_ < 0.001; UA: 323.7± 86.2 vs 358.5 ± 93.2, *P*_01_ < 0.001); no significant improvements were observed at 24 w post-treatment (eGFR: 86.7 ± 20.4 vs 81.4 ± 18.6, *P*_02_ = 0.013; Scr: 83.6 ± 17.9 vs 87.9 ± 18.3, *P*_02_ = 0.014; UA: 320.8 ± 76.3 vs 349.3 ± 91.0, *P*_02_ = 0.004). When the patients were grouped by liver conditions, non-cirrhotic patients and cirrhotic patients had decreased eGFR levels and increased Scr levels at the end of treatment; at 24 w post-treatment, the eGFR and Scr levels were significantly improved in non-cirrhotic patients (88.4 ± 21.7 vs 83.8 ± 18.5, *P*_02_ = 0.142; 84.4 ± 20.4 vs 87.0 ± 16.9, *P*_02_ = 0.088), while no obvious improvements were observed in cirrhotic patients (84.3 ± 18.7 vs 78.1 ± 18.6, *P*_02_ = 0.002; 83.2 ± 17.7 vs 89.2 ± 20.6, *P*_02_ = 0.006). Clinical physicians should closely monitor renal function in patients treated with SOF-containing regimens, especially in cirrhotic patients.

## INTRODUCTION

Hepatitis C virus (HCV) is an important pathogen affecting approximately 130‒150 million people worldwide [1, 2]. Chronic hepatitis C (CHC) patients have enhanced risk of cirrhosis and hepatocellular carcinoma [3, 4]. The emergence of direct-acting antivirals (DAAs) has revolutionized the treatment of HCV with shorter treatment durations, higher sustained virological response (SVR) rates, fewer adverse events (AEs) and fewer contraindications than those of traditional peginterferon and ribavirin (PegIFN/RBV, PR) treatment regimens [5‒8].

With the wide application of DAAs, challenging issues regarding the efficacy and safety of new DAAs regimens have arisen, e.g., resistance-associated variants, drug-drug interactions (DDIs), HBV (hepatitis B virus) reactivation, hepatotoxicity and nephrotoxicity [9‒20]. In October 2016, the United States Food and Drug Administration issued a black box warning regarding the risk for HBV activation with 9 DAAs, citing 24 cases that included 3 reports of acute liver failure (https://www.fda.gov/). The Institute for Safe Medicine Practices followed up with a review of Adverse Event Reporting System data covering a 12-month span. The review uncovered 524 cases of liver failure associated with DAAs and that 31.5% of the patients had died at the time of the review (http://www.ismp.org/ default.asp). Traditional PR treatment regimens and first-generation protease inhibitors are considered nephrotoxic [21, 22]. Although all-oral DAAs regimens were well tolerated in clinical trials, recent real-world studies demonstrated some cases with nephrotoxicity that were treated with sofosbuvir (SOF)-containing regimens [18‒20]. Some cases with hepatotoxicity and nephrotoxicity associated with DDIs were reported in CHC patients with concomitant diseases, HBV or HIV co-infections, and liver transplantations [11, 14, 16, 17].

Considering the increasing occurrence of cases with hepatotoxicity, nephrotoxicity, and DDIs, this study aimed to explore the changes of hepatic and renal function indices in CHC patients treated with DAAs.

## RESULTS

### Baseline characteristics and treatment efficacy

The main demographic, virological and clinical characteristics are described in Table 1. Of the 43 patients, 51.2% were more than 60 years old, with a mean age of 57.9 ± 15.7 years; 55.8% were male; 46.5% were PR treatment experienced; and 41.9% had cirrhosis. The mean values for estimated glomerular filtration rate (eGFR), serum creatinine (Scr) and uric acid (UA) were 86.7 ± 20.4 ml/min/1.73 m^2^, 83.9 ± 19.1 μmol/L, and 323.7 ± 86.2 μmol/L, respectively. Compared with non-cirrhotic patients, cirrhotic patients had higher PR treatment-experience rates (24% vs 77.8%, *P* = 0.001), mean liver stiffness measurement (LSM) scores (8.0 ± 3.7 vs 29.9 ± 14.6, *P* < 0.001), and aminotransferase (AST) levels (41.6 ± 24.2 vs 69.4 ± 34.4, *P* = 0.003) and lower platelet (PLT) counts (179.2 ± 61.5 vs 92.1 ± 31.7, *P* < 0.001). Other baseline characteristics did not differ significantly between non-cirrhotic patients and cirrhotic patients (Table 1). One patient discontinued the SOF/daclatasvir (DAC) treatment 8 w after the initiation of the treatment due to the development of renal area pain; all other patients completed the treatment and follow-up. A total of 97.7% (42/43) of the patients achieved SVR at 12 w post-treatment (SVR 12); one non-cirrhotic patient treated with SOF/ledipasvir (LDV) still had a detectable HCV RNA at the end of treatment.

**Table 1:** Baseline characteristics of enrolled patients

Characteristics	All (*n* = 43)	non-Cirrhotic (*n*_1_ = 25)	Cirrhotic (*n*_2_ = 18)	*P*(*n*_1_ vs *n*_2_)
Age (mean)	57.9 ± 15.7	54.9 ± 17.7	62.1 ± 11.8	NS
Age (> 60 years)	22 (51.2%)	10 (40%)	12 (66.7%)	NS
Male	24 (55.8%)	15 (60%)	9 (50%)	NS
HCV RNA log_10_ (IU/ml)	6.64 ± 0.87	6.81 ± 0.59	6.40 ± 1.13	NS
PR (experienced)	20 (46.5%)	6 (24%)	14 (77.8%)	0.001
LSM (kPa)	17.2 ± 14.6	8.0 ± 3.7	29.9 ± 14.6	< 0.001
ALT (IU/L)	57.8 ± 38.1	54.0 ± 40.5	63.2 ± 35.0	NS
AST (IU/L)	53.2 ± 31.8	41.6 ± 24.2	69.4 ± 34.4	0.003
PLT (10^9^/L)	142.7 ± 66.8	179.2 ± 61.5	92.1 ± 31.7	< 0.001
eGFR(ml/min/1.73 m^2^)	86.7 ± 20.4	88.4 ± 21.7	84.3 ± 18.7	NS
Scr (μmol/L)	83.9 ± 19.1	84.4 ± 20.4	83.2 ± 17.7	NS
UA (μmol/L)	323.7 ± 86.2	318.7 ± 83.9	330.7 ± 91.2	NS
BUN (mmol/L)	5.23 ± 1.46	4.97 ± 1.27	5.60 ± 1.67	NS

NS: no significance.

### Changes of hepatic function indices

**Figure 1:**
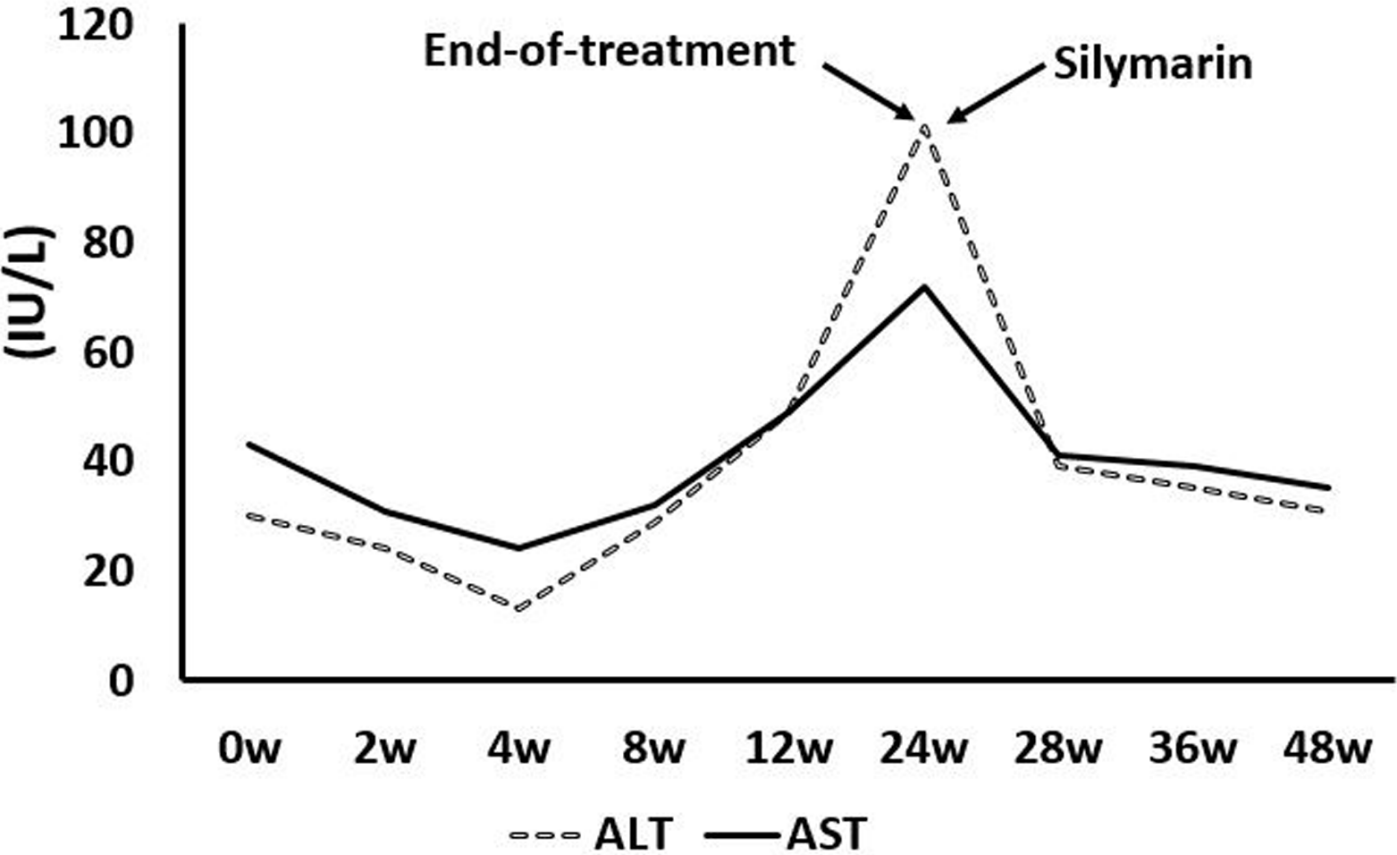
One decompensated cirrhotic patient with liver injury associated with treatment with DAAs

The mean aminotransferase (ALT) and AST levels at the end of treatment and at 24 w post-treatment were significantly decreased compared with the baseline levels (ALT: 57.8 ± 38.1 vs 19.8 ± 14.2, *P*_01_ < 0.001; 57.8 ± 38.1 vs 17.3 ± 6.8, *P*_02_<0.001; AST: 53.2 ± 31.8 vs 24.4 ± 9.9, *P*_01_ < 0.001; 53.2 ± 31.8 vs 22.7 ± 6.9, *P*_02_ < 0.001) (Table 2), whereas one decompensated cirrhotic patient (LSM = 41.2 kPa, mild ascites) treated with SOF/LDV developed a liver injury at the end of treatment (ALT = 101 IU/L; AST = 72 IU/L). A 53-year-old female patient was hospitalized with a persistent low-grade fever, fatigue and sleepiness on January 22nd, 2016. The baseline ALT and AST levels were within the normal ranges and there was neither alcohol use nor concomitant medications during the SOF/LDV treatment. Silymarin capsules (MADAUS GmbH, Germany) were prescribed for two weeks (140 mg/bid), and ALT and AST levels were kept in normal ranges until 24 weeks post-treatment (Figure 1). Along with the recovery of the liver function, the PLT count at 24 w post-treatment was significantly increased compared with the PLT count at baseline (142.7 ± 66.8 vs 148.8 ± 67.4, *P*_01_ = 0.112; 142.7 ± 66.8 vs 155.7 ± 66.9, *P*_02_ < 0.01) (Table 2).

**Table 2:** Changes of hepatic and renal function indices among different time points

	T0	T1	T2	P_01_	P_02_	PDAAs*Time
ALT (IU/L)	57.8 ± 38.1	19.8 ± 14.2	17.3 ± 6.8	< 0.001	< 0.001	NS
AST (IU/L)	53.2 ± 31.8	24.4 ± 9.9	22.7 ± 6.9	< 0.001	< 0.001	NS
PLT (10^9^/L)	142.7 ± 66.8	148.8 ± 67.4	155.7 ± 66.9	NS	0.003	NS
eGFR(ml/min/1.73 m2)	86.7 ± 20.4	80.5 ± 21.3	81.4 ± 18.6	0.005	0.013	NS
Scr (μmol/L)	83.9 ± 19.1	89.6 ± 21.1	87.9 ± 18.3	< 0.001	0.014	NS
UA (μmol/L)	323.7 ± 86.2	358.5 ± 93.2	349.3 ± 91.0	< 0.001	0.004	NS
BUN (mmol/L)	5.23 ± 1.46	5.29 ± 1.53	5.72 ± 1.81	NS	0.017	NS

T0: baseline; T1: end of treatment; T2: 24 w after the end of treatment. *P*_01_: significance of difference between T0 and T1; *P*_02_: significance of difference between T0 and T2; *P*_DAAs*Time_: interactive effects of DAAs regimens and time points on the changes of renal function indices. NS: no significance.

### Changes in renal function indices

**Figure 2:**
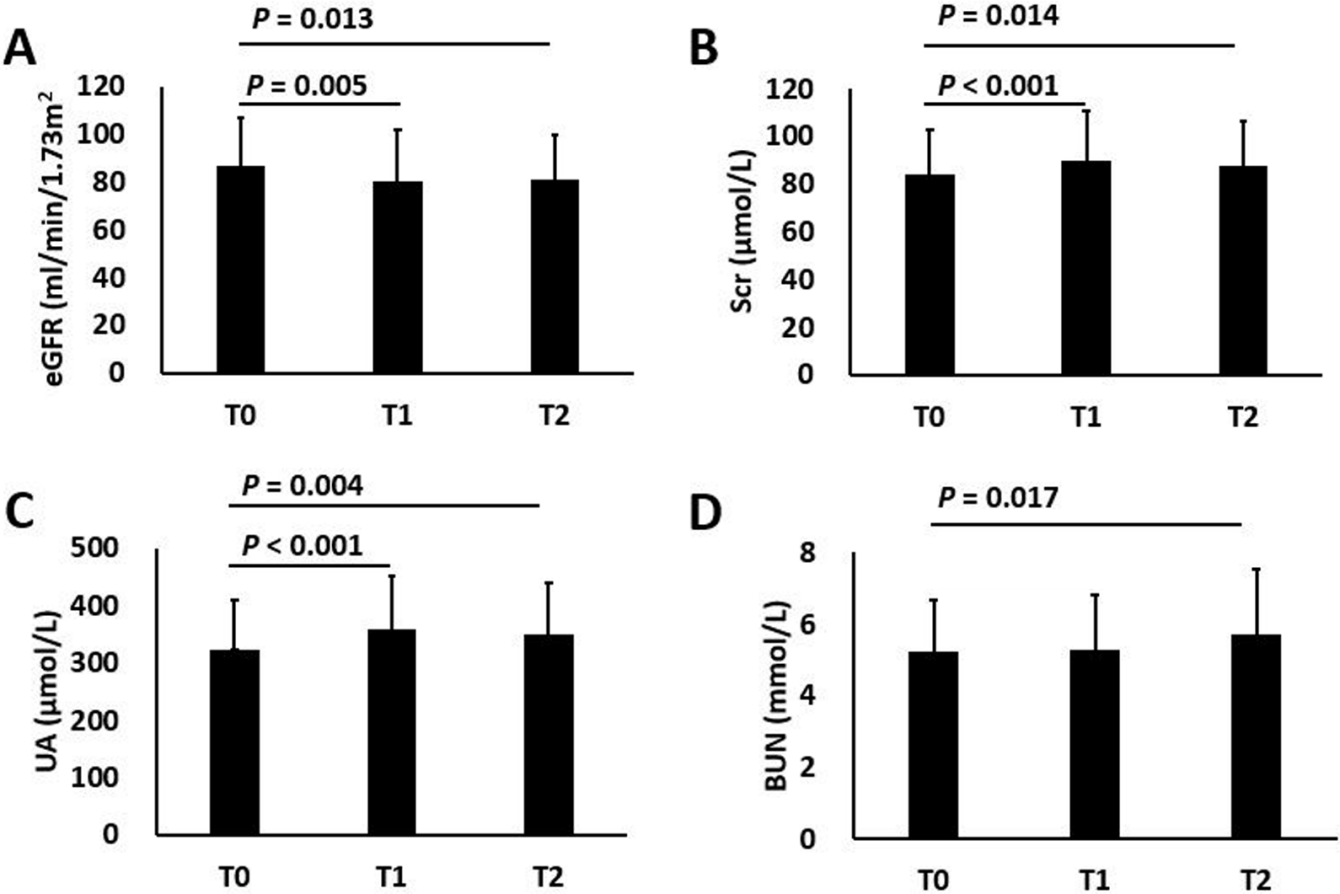
Changes in renal function indices among different observing points (**A**) eGFR; (**B**) Scr; (**C**) UA; (**D**) BUN. T0: baseline; T1: end of treatment; T2: 24 w post-treatment. *P*_01_: statistical significance of the difference between T0 and T1; *P*_02_: statistical significance of the difference between T0 and T2.

At the end of treatment, the eGFR level was significantly decreased and the Scr and UA levels were significantly increased compared with baseline levels (eGFR: 86.7 ± 20.4 vs 80.5 ± 21.3, *P*_01_ = 0.005; Scr: 83.9 ± 19.1 vs 89.6 ± 21.1, *P*_01_ < 0.001; UA: 323.7 ± 86.2 vs 358.5 ± 93.2, *P*_01_ < 0.001), and no significant improvements were observed at 24 w post-treatment (eGFR: 86.7 ± 20.4 vs 81.4 ± 18.6, *P*_02_ = 0.013; Scr: 83.6 ± 17.9 vs 87.9 ± 18.3, *P*_02_ = 0.014; UA: 320.8 ± 76.3 vs 349.3 ± 91.0, *P*_02_ = 0.004). The blood urea nitrogen (BUN) level at the end of treatment showed no significant changes compared with the baseline level, while an increased BUN level was observed 24 w post-treatment (5.23 ± 1.46 vs 5.72 ± 1.81, *P*_02_ = 0.017) (Figure 2). The DAAs regimens and the time points had no interactive effects on the changes in renal function indices (*P*_DAAs*Time_ > 0.05), which indicated that the SOF/DAC and SOF/LDV regimens had the same effects on the changes in renal function indices with main effects (Table 2).

**Figure 3:**
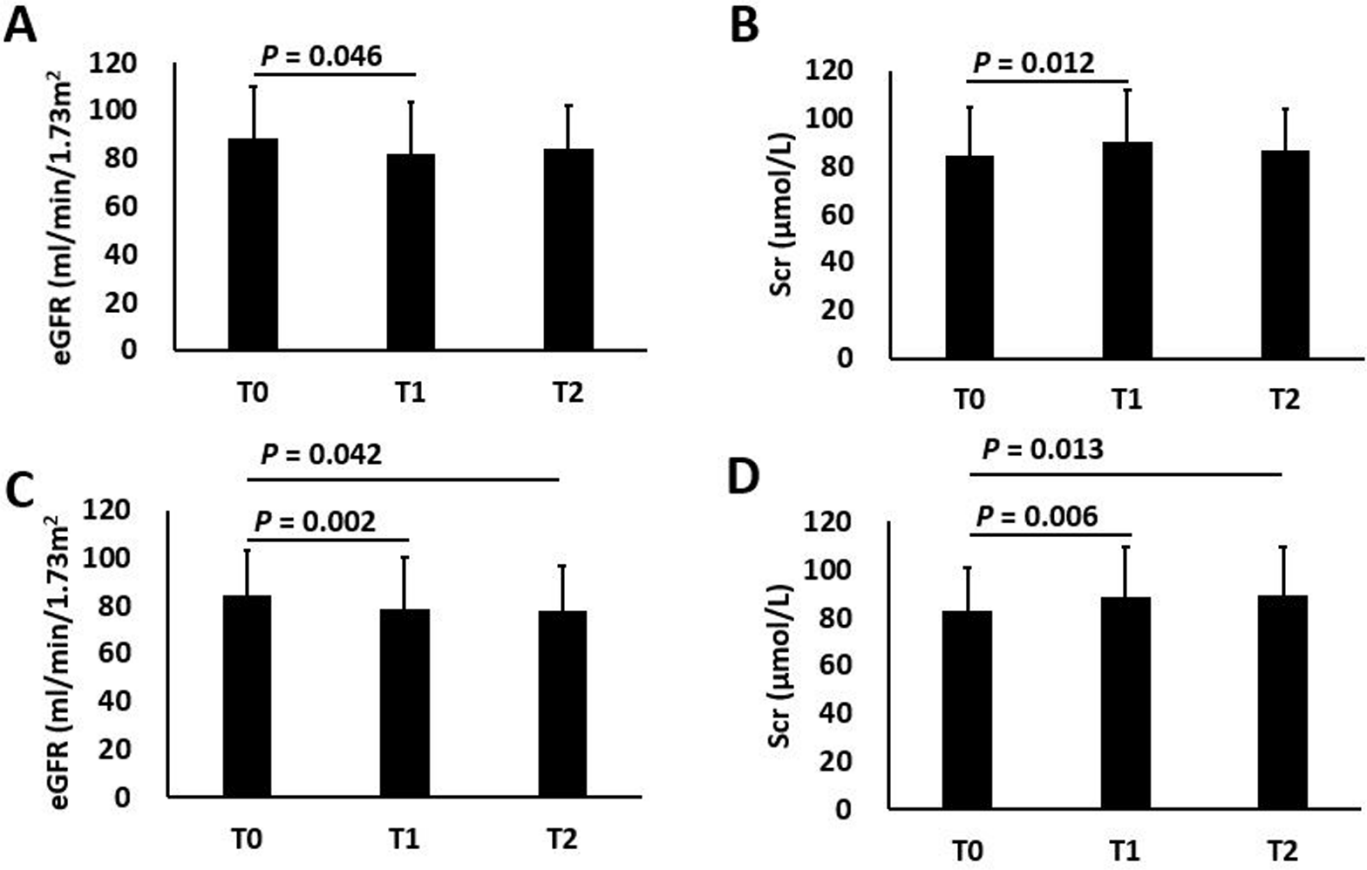
Changes of eGFR and Scr levels in non-cirrhotic and cirrhotic patients Changes of eGFR (**A**) and Scr (**B**) levels in non-cirrhotic patients; Changes of eGFR (**C**) and Scr (**D**) levels in cirrhotic patients; T0: baseline; T1: end of treatment; T2: 24 w post-treatment. *P*_01_: statistical significance of the difference between T0 and T1; *P*_02_: statistical significance of the difference between T0 and T2.

Considering the difference in liver conditions and treatment durations, changes in renal function indices were compared between non-cirrhotic patients and cirrhotic patients (Table 3). At the end of treatment, non-cirrhotic patients and cirrhotic patients had decreased eGFR levels and increased Scr levels (non-cirrhotic: 88.4 ± 21.7 vs 81.9 ± 21.4, *P*_01_ = 0.046; 84.4 ± 20.4 vs 90.1 ± 22.0, *P*_01_ = 0.012; cirrhotic: 84.3 ± 18.7 vs 78.7 ± 21.5, *P*_01_ = 0.042; 83.2 ± 17.7 vs 88.9 ± 20.3, *P*_01_ = 0.013); at 24 w post-treatment, the eGFR and Scr levels were significantly improved in non-cirrhotic patients (eGFR: 88.4 ± 21.7 vs 83.8 ± 18.5, *P*_02_ = 0.142; Scr: 84.4 ± 20.4 vs 87.0 ± 16.9, *P*_02_ = 0.088), while no obvious improvements were observed in cirrhotic patients (eGFR: 84.3 ± 18.7 vs 78.1 ± 18.6, *P*_02_ = 0.002; Scr: 83.2 ± 17.7 vs 89.2 ± 20.6, *P*_02_ = 0.006) (Figure 3). The UA levels at the end of treatment and at 24 w post-treatment were significantly increased compared with baseline levels (non-cirrhotic: 318.7 ± 83.9 vs 355.2 ± 84.4, *P*_01_ = 0.001; 318.7 ± 83.9 vs 344.2 ± 92.2, *P*_02_ = 0.031; cirrhotic: 330.7 ± 91.2 vs 363.2 ± 106.6, *P*_01_ = 0.017; 330.7 ± 91.2 vs 356.5 ± 91.3, *P*_02_ = 0.04). The BUN levels in non-cirrhotic and cirrhotic patients had no significant changes across time points (Table 3).

**Table 3:** Changes of renal function indices in non-cirrhotic and cirrhotic patients

		T0	T1	T2	*P*_01_	*P*_02_
non-Cirrhotic	eGFR	88.4 ± 21.7	81.9 ± 21.4	83.8 ± 18.5	0.046	NS
(*N* = 25)	Scr	84.4 ± 20.4	90.1 ± 22.0	87.0 ± 16.9	0.012	NS
	UA	318.7 ± 83.9	355.2 ± 84.4	344.2 ± 92.2	0.001	0.031
	BUN	4.97 ± 1.27	4.97 ± 1.19	5.38 ± 1.52	NS	NS
Cirrhotic	eGFR	84.3 ± 18.7	78.7 ± 21.5	78.1 ± 18.6	0.042	0.002
(*N* = 18)	Scr	83.2 ± 17.7	88.9 ± 20.3	89.2 ± 20.6	0.013	0.006
	UA	330.7 ± 91.2	363.2 ± 106.6	356.5 ± 91.3	0.017	0.04
	BUN	5.60 ± 1.67	5.73 ± 1.84	6.20 ± 2.11	NS	NS

T0: baseline; T1: end of treatment; T2: 24 w after the end of treatment. P01: significance of difference between T0 and T1; P02: significance of difference between T0 and T2. NS: no significance.

### Comorbidities and concomitant medications

A total of 62.7% (27/43) of the patients were found to have comorbidities; the main comorbidities were hypertension (32.6%), hyperlipidemia (16.3%), gastrointestinal diseases (14.0%), diabetes mellitus (11.6%), and thyroid dysfunction (11.6%). A total of 74.4% (32/43) of the patients used at least one concomitant medication; the main medications were antihypertensives (32.6%), antibiotics (23.3%), antipyretic analgesics (20.9%), statins (14.0%), antidiabetics (11.6%), gastro-kinetic agents (11.6%), proton pump inhibitors (9.3%), and hepatoprotectants (9.3%).

## DISCUSSION

The availability and rapid development of DAAs has revolutionized the management of CHC and achieved a high SVR rate with a low incidence of AEs. Although DAAs are highly effective and well tolerated, some cases with hepatotoxicity and nephrotoxicity have been reported [14‒20]. Thus, this study analyzed the efficacy of DAAs treatment and the effects on hepatic and renal function indices in CHC patients in clinical practice.

In this study, combined DAA therapy achieved a promising SVR rate (97.7%) that was not significantly different from the rates reported in previous studies [23‒26]. However, the abnormal changes in renal function indices were unexpected. At the end of treatment, the eGFR level was significantly decreased and the Scr and UA levels were significantly increased; at 24 w post-treatment, non-cirrhotic patients showed improvements, whereas a persistent decrease in eGFR level and increases in Scr and UA levels were observed in cirrhotic patients (Table 2, Table 3). eGFR and Scr were important indices for assessing renal function, and an elevated UA level also predicted a rapid decline in kidney function [27].

Although the specific mechanisms were unknown, possible reasons for the abnormal changes in renal function indices in this study are as follows: (1) Potential DDIs caused by complicated concomitant medication use might be a major reason for the abnormal changes. Each DAA had its own metabolism and potential DDIs [28]; drug metabolic enzymes, such as cytochrome P450 (CYP450); drug transporters, such as P-glycoprotein (P-gp); and breast cancer resistance protein (BCRP) were the most common pathways leading to DDIs. Unlike in clinical trials, concomitant medications that had potential DDIs with DAAs are frequently prescribed to patients with chronic HCV infection in clinical practice [29, 30]. DAAs or concomitant medications could act as substrates, inhibitors and inducers of metabolic enzymes and transporters, leading to an elevated blood drug concentration [31, 32]. In this study, two combined DAAs regimens had the same effects on changes in renal function indices, and we speculated that the SOF use in the two regimens might account for the abnormal changes in renal function indices. SOF is intracellularly metabolized and forms the active metabolite GS-461203 and the inactive compound GS-331007, which is primarily renally excreted; moreover, SOF is a substrate of P-gp and BCRP and is 61–65% bound to plasma proteins [33]. DDIs among SOF, GS-331007 and concomitant medications could cause kidney injury due to increased blood drug concentration, especially when concomitant medications with potential hepatotoxicity and nephrotoxicity are prescribed. (2) High frequencies of comorbidities and concomitant medications in this elderly patients might also contribute to the abnormal changes in renal function indices. This was a relatively elderly cohort; the mean age was 57.9 ± 15.7 years, with 51.2% of the patients were more than 60 years old, and 41.9% of the patients had cirrhosis (Table 1); 62.7% of the patients were found to have comorbidities and 74.4% of the patients had at least one concomitant medication, including antihypertensives (32.6%), antibiotics (23.3%), antipyretic analgesics (20.9%), statins (14.0%), antidiabetics (11.6%), gastro-kinetic agents (11.6%), proton pump inhibitors (9.3%), or hepatoprotectants (9.3%). Recent studies also showed high frequencies of comorbidities and concomitant medications in elderly patients during treatment with DAAs, many of which had potential DDIs with DAAs [34‒36]. (3) Cirrhotic patients had persistent abnormal changes in renal function indices. Cirrhotic patients received 24 weeks of combined DAA treatment, and prolonged treatment durations increased the risk of DDIs between DAAs and concomitant medications; the cirrhotic patients were older than the non-cirrhotic patients (Table 1), elderly patients had more concomitant medications, which represented high risks for DDIs [34, 35] and eGFR declined with aging by approximately 1 mL/min/1.73 m^2^ annually which could result in a decreased renal elimination capacity and increased blood drug concentration of DAAs or concomitant medications [27, 37]; studies using a single 400 mg dose of SOF in patients with renal impairment have shown a significant increase in serum levels of SOF and the metabolite GS331007 compared with levels in patients with normal renal function (eGFR > 80 ml/min/1.73 m^2^) [33]; cirrhotic patients in this cohorts had relatively lower eGFR levels than non-cirrhotic patients (Table 1), which might result in a decreased renal elimination capacity and the accumulation of SOF and its metabolite GS331007 or concomitant medications; these factors might have jointly contributed to the persistent elevation of Scr and UA levels in cirrhotic patients.

One decompensated cirrhotic patient treated with SOF/LDV was found to have elevated ALT and AST levels, and there were no other virus infections, alcohol use, or concomitant medications (Figure 1). Cirrhosis destroyed the structure and function of the liver, reduced the expression of drug metabolizing enzymes and thus reduced metabolizing capacity, which increased the blood concentration of the drugs metabolized by these enzymes [33, 38]; this might have led to liver injury in the patient with decompensated cirrhosis.

The abnormal changes observed in renal function indices associated with the SOF-containing regimens in this study should be taken as a note of caution. Clinical physicians should closely monitor hepatic and renal function in patients treated with SOF-containing regimens, especially in cirrhotic patients.

## MATERIALS AND METHODS

### Patients

Forty-three CHC patients who were treated with DAAs while hospitalized in Peking University First Hospital between January 2015 and January 2017 and met the following criteria were enrolled in this study: (1) infected with HCV GT 1b; (2) negative for hepatitis A virus immunoglobulin M (HAV IgM), hepatitis B surface antigen (HBsAg), hepatitis E virus IgM (HEV IgM), human immunodeficiency virus (HIV), Epstein-Barr virus (EBV), and cytomegalovirus (CMV); (3) no severe renal function impairment (eGFR < 30 ml/min/1.73 m^2^) and end-stage renal disease; (4) no severe heart disease; (5) no active drug use and no alcohol use; (6) no pregnancy; (7) DAAs treatment regimens prescribed according to the recommendation of the new guidelines [39‒41]; and (8) clinical information is intact. A total of 31 patients were excluded, including 14 HCV GT2a infected patients, one HBV/HCV co-infected patient, one CMV/HCV co-infected patient, 3 patients with severe renal function damage, one patient treated with DAAs regimens who did not comply with the guidelines, and 11 patients with incomplete clinical information. Of the 43 patients, 18 were treated with SOF (400 mg/day) / DAC (60 mg/day) and 25 were treated with SOF (400 mg/day) / LDV (90 mg/day); non-cirrhotic patients were treated for 12 weeks and cirrhotic patients were treated for 24 weeks. All study participants provided informed written consent prior to enrollment in the study. Ethical approval was given by the Ethics Committee. The study was in compliance with the Helsinki Declaration.

### Clinical data collection, HCV RNA quantitation, and genotyping

Hematological, biochemical, and urine tests were performed and recorded at 0 w, 2 w, 4 w, 8 w, 12 w, or 24 w during the DAA treatment, as well as at 4 w, 12 w, and 24 w post-treatment at a clinical laboratory. The virological endpoint was the achievement of SVR 12, and clinical indices at 24 w post-treatment were recorded as clinical endpoints. The ALT, AST and PLT count were used to assess liver function; eGFR, Scr, UA, and BUN were used to assess renal function.

LSMs were measured by transient elastography (Fibroscan, Echosens, Paris), and the presence of cirrhosis was determined by LSM ≥ 17.6 kPa [42–44]. The eGFR was calculated with the Modification of Diet in Renal Disease Study equation adjusted for the Chinese population: eGFR = 175 *(serum creatinine)^−1.234^ *age^−0.179^ *0.79 (if female) [45].

HCV RNA quantitation and genotyping were measured at the virus laboratory in the department of infectious disease. Serum HCV RNA quantitation was measured using a COBAS Taqman HCV Test kit (Roche Molecular Systems Inc., Pleasanton, CA, USA); a COBAS AmpliPrep instrument was used for automated specimen processing, and a COBAS Taqman analyzer was used for automated amplification and detection [46]. The detailed detection procedures were performed according to the manufacturer’s instructions. HCV genotypes were determined by restriction fragment length polymorphism (RFLP) analysis of the amplified 5′-noncoding genome region [47]. Detailed procedures were performed according to the following protocol: HCV RNA was extracted from 140 μL serum samples using a QIAamp viral RNA mini kit (Qiagen, Hilden, Germany); reverse transcription and polymerase chain reaction (PCR) amplification were conducted using BG1 (5′-CTGTGAGGAACTACTGTCTT-3′) and BG2 (5′-AACACTACTCGGCTAG CAGT-3′) as upstream and downstream primers for the first round reaction and BG3 (5′-TTCACGCAGAAAGCGTCTAG-3′) and BG4 (5′-GTTGATCCA AGAAAGGACCC-3′) as upstream and downstream primers for the second round reaction; the PCR products were purified using a QIAquick PCR Purification Kit (Qiagen, Hilden, Germany) and digested with Hae III at 37°C for 2 hours; and then, agarose gel electrophoresis was performed to analyze the RFLP of the digestion products.

### Statistical analysis

Microsoft Excel (Microsoft, Redmond, Washington, USA) was used for data collection and analyses. Continuous variables were expressed as the means ± standard deviations and compared using Student’s *t*-test or Fisher’s exact test; categorical variables were expressed as absolute numbers and percentages and were compared using the Chi-square test. Repeated measures analysis of variance was used to provide comparisons between different time points and different groups and to calculate the interaction effects between regimens of DAAs and time points. Mauchly’s test of sphericity was used to judge whether there were relations among the repeatedly measured data. If any *P* < 0.05, the Greenhouse-Geisser corrected results were utilized; Bonferroni or Fisher’s Least Significant Difference tests (when Epsilon < 0.7, Bonferroni test) were used to examine pairwise comparisons of the repeatedly measured data at different measurement times. We conducted the statistical analyses using SPSS version 16.0. *P* < 0.05 was considered to indicate statistical significance.

## Abbreviations

CHC: chronic hepatitis C
DAAs: directly acting antivirals
eGFR: estimated glomerular filtration rate
Scr: serum creatinine
UA: uric acid
HCV: hepatitis C virus
HBV: hepatitis C virus
SVR: sustained virological response
AEs: adverse events
PR: peginterferon and ribavirin
DDIs: drug-drug interactions
SOF: sofosbuvir
DAC: daclatavir
LDV: ledipasvir
LSM: liver stiffness measurement
RFLP: restriction fragment length polymorphism
